# Supplementary material for: Novel data show expert wildlife agencies are important to endangered species protection
Source: Nat Commun. 2019 Aug 1;10:3467. doi: 10.1038/s41467-019-11462-9 (PMC6671991; doi:10.1038/s41467-019-11462-9)
Supplement: Supplementary file 1 — Supplementary Information [file 41467_2019_11462_MOESM1_ESM.pdf]

Supplementary Information

Novel data show expert wildlife agencies are central to endangered species protection.

Evans et al.

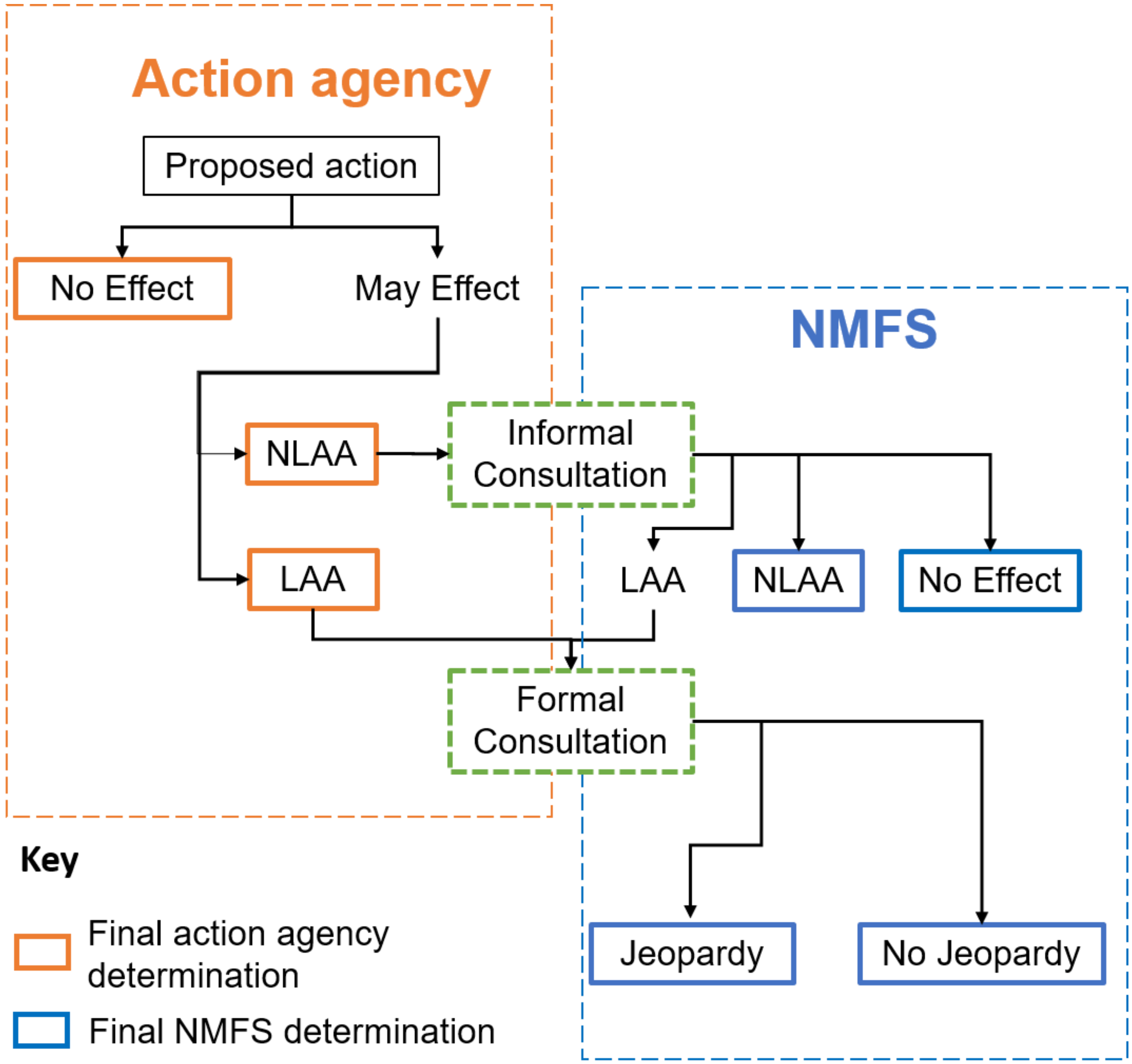

Supplementary Figure 1. Conceptual diagram displaying possible outcomes of consultation between federal action agencies and the National Marine Fisheries Service (NMFS). Action agencies and NMFS can both determine that proposed actions will have 'No Effect,' or may affect but are 'Not Likely to Adversely Affect' (NLAA) listed species. If either determines an action is 'Likely to Adversely Affect' (LAA) listed species, NMFS must make a 'Jeopardy' or 'No Jeopardy' determination. NMFS cannot issue an LAA final determination, and action agencies do not make 'Jeopardy' or 'No Jeopardy' determinations.

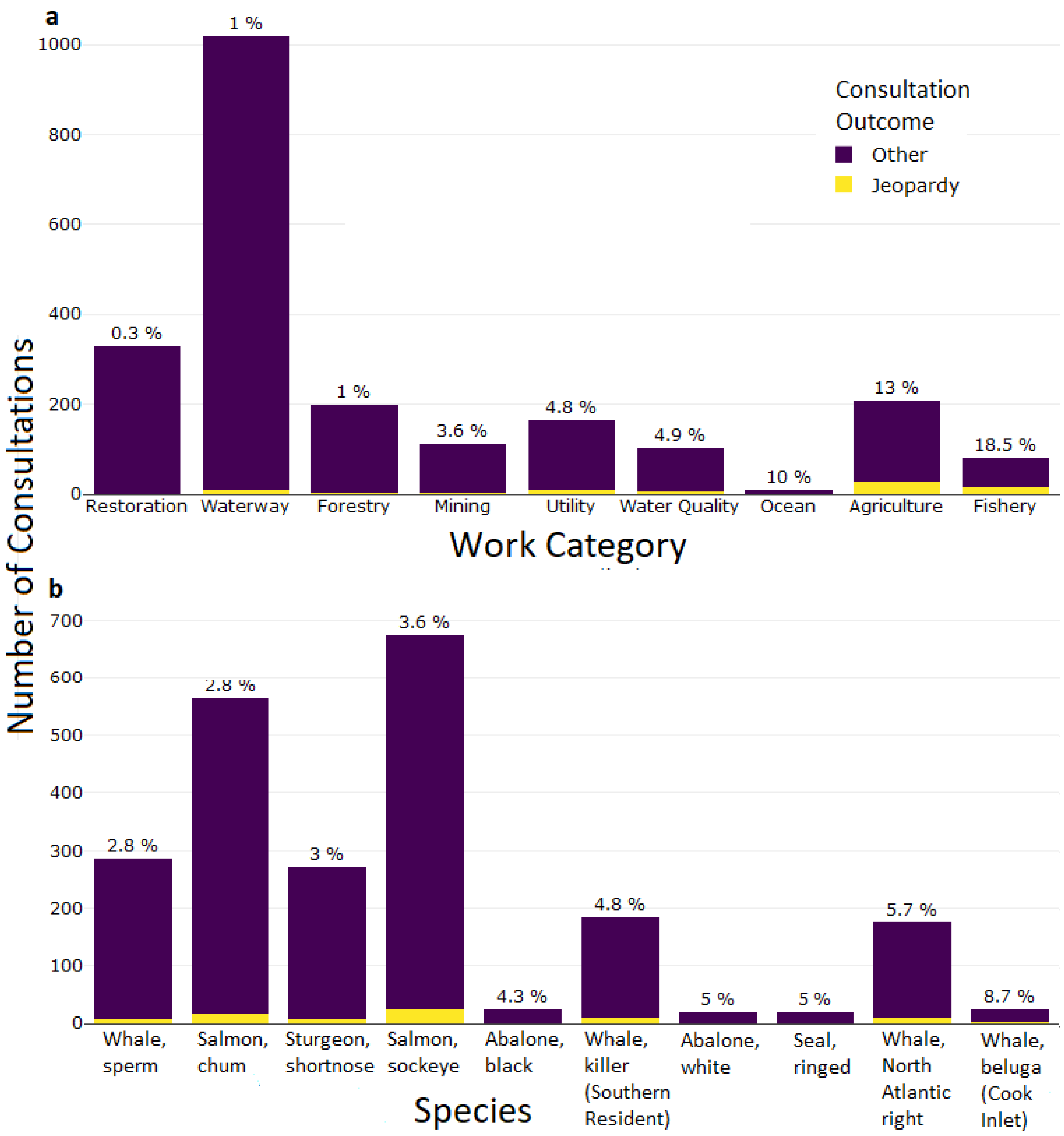

Supplementary Figure 2. Jeopardy conclusion rates among work categories (**a**) and jeopardy determination rates among species (**b**) in formal U.S. Endangered Species Act section 7 consultations between U.S federal agencies and the National Marine Fisheries Service from 2000 to 2017. Shown are the ten members of each group, involved in at least 10 consultations, with the highest rates and the percentage of jeopardy conclusions for each member. Jeopardy conclusions were rare, even among the most frequently jeopardized species.
