# Supplementary material for: Novel data show expert wildlife agencies are important to endangered species protection
Source: Nat Commun. 2019 Aug 1;10:3467. doi: 10.1038/s41467-019-11462-9 (PMC6671991; doi:10.1038/s41467-019-11462-9)
Supplement: Supplementary file 3 — Reporting Summary [file 41467_2019_11462_MOESM3_ESM.pdf]

nature research

Corresponding author(s): *Double-blind peer review submissions: write DBPR and your manuscript number here instead of author names.*

Reporting Summary

Nature Research wishes to improve the reproducibility of the work that we publish. This form provides structure for consistency and transparency in reporting. For further information on Nature Research policies, see Authors & Referees and the Editorial Policy Checklist.

Statistical parameters

When statistical analyses are reported, confirm that the following items are present in the relevant location (e.g. figure legend, table legend, main text, or Methods section).

n/a	Confirmed	
☐	☒	The exact sample size (n) for each experimental group/condition, given as a discrete number and unit of measurement
☒	☐	An indication of whether measurements were taken from distinct samples or whether the same sample was measured repeatedly
☐	☒	The statistical test(s) used AND whether they are one- or two-sided<br>*Only common tests should be described solely by name; describe more complex techniques in the Methods section.*
☐	☒	A description of all covariates tested
☐	☒	A description of any assumptions or corrections, such as tests of normality and adjustment for multiple comparisons
☐	☒	A full description of the statistics including central tendency (e.g. means) or other basic estimates (e.g. regression coefficient) AND variation (e.g. standard deviation) or associated estimates of uncertainty (e.g. confidence intervals)
☐	☒	For null hypothesis testing, the test statistic (e.g. F, t, r) with confidence intervals, effect sizes, degrees of freedom and P value noted<br>*Give P values as exact values whenever suitable.*
☒	☐	For Bayesian analysis, information on the choice of priors and Markov chain Monte Carlo settings
☒	☐	For hierarchical and complex designs, identification of the appropriate level for tests and full reporting of outcomes
☐	☒	Estimates of effect sizes (e.g. Cohen's d, Pearson's r), indicating how they were calculated
☒	☐	Clearly defined error bars<br>*State explicitly what error bars represent (e.g. SD, SE, CI)*

Our web collection on statistics for biologists may be useful.

Software and code

Policy information about availability of computer code

Data collection	Data was provided in csv format by the National Marine Fisheries Service from the Public Consultation Tracking Database.
Data analysis	All statistical analyses were performed in R version 3.5.1 using the cooccur, and vegan packages. All code used in data cleaning and analysis is maintained in a GitHub repository (https://github.com/mjevans26/NMFS7). A static version is available through an Open Science Framework repository (DOI: 10.17605/OSF.IO/UCFKJ)

For manuscripts utilizing custom algorithms or software that are central to the research but not yet described in published literature, software must be made available to editors/reviewers upon request. We strongly encourage code deposition in a community repository (e.g. GitHub). See the Nature Research guidelines for submitting code & software for further information.

Data

Policy information about availability of data

All manuscripts must include a data availability statement. This statement should provide the following information, where applicable:

- Accession codes, unique identifiers, or web links for publicly available datasets
- A list of figures that have associated raw data
- A description of any restrictions on data availability

The consultation data that support the findings of this study, and all R code used to conduct statistical analyses and create graphs are available in a public Open Science Framework repository (DOI: 10.17605/OSF.IO/UCFKJ).

Field-specific reporting

Please select the best fit for your research. If you are not sure, read the appropriate sections before making your selection.

☐ Life sciences ☐ Behavioural & social sciences ☒ Ecological, evolutionary & environmental sciences

For a reference copy of the document with all sections, see nature.com/authors/policies/ReportingSummary-flat.pdf

Ecological, evolutionary & environmental sciences study design

All studies must disclose on these points even when the disclosure is negative.

Study description	This study evaluates the implementation of section 7 consultations between federal agencies and the National Marine Fisheries Service under the US Endangered Species Act. We calculate summary statistics describing the frequency with which listed species, federal agencies and work types are involved in consultation. We estimate measures of association among these categorical variables and departures from random distributions of outcomes of consultations using randomization procedures. Additionally, we estimate the degree of agreement of the effects of proposed federal actions on listed species between the agencies proposing those actions and NMFS. This research did not involve any experimentation or manipulation.
Research sample	We analyzed all consultation data from the National Marine Fisheries Service recorded from 2000 - 2017. These data include information regarding the species, federal agencies, proposed actions, and outcomes involved in section 7 consultations under the US Endangered Species Act. This dataset is stored in the Public Consultation Tracking System, and was provided to the authors by the National Oceanic and Atmospheric Administration's Office of Protected Resources.
Sampling strategy	We did not perform sampling, instead analyzing the entirety of consultation records.
Data collection	Consultation data is recorded by NMFS biologists during consultation. These data are stored in the Public Consultation Tracking System.
Timing and spatial scale	Consultation data is and was collected continuously as section 7 consultations were conducted as mandated by the ESA whenever federal actions are judged to potentially affect species on the Endangered Species List. Consultations occur within and across the United States and its territories.
Data exclusions	We excluded data collected before 2000, as stated in the manuscript. This decision was made after identifying a substantial decline in the number of records prior to 2000. In conjunction with biologists at NMFS, we determined records prior to 2000 were likely incomplete.
Reproducibility	We have provided all data and code used in our analyses in public Open Science Framework repositories, to allow other researchers to reproduce our results.
Randomization	Our study did not involve treatments or experimental groups.
Blinding	The data analyzed was collected previously by NMFS biologists. We use a unique hash digest to make this personnel information anonymous.

Did the study involve field work? ☐ Yes ☒ No

Reporting for specific materials, systems and methods

Materials & experimental systems

n/a	Involved in the study
☒	☐ Unique biological materials
☒	☐ Antibodies
☒	☐ Eukaryotic cell lines
☒	☐ Palaeontology
☒	☐ Animals and other organisms
☒	☐ Human research participants

Methods

n/a	Involved in the study
☒	☐ ChIP-seq
☒	☐ Flow cytometry
☒	☐ MRI-based neuroimaging
